# Correction: 15-keto-prostaglandin E_2_ activates host peroxisome proliferator-activated receptor gamma (PPAR-γ) to promote *Cryptococcus neoformans* growth during infection

**DOI:** 10.1371/journal.ppat.1009058

**Published:** 2020-11-04

**Authors:** Robert J. Evans, Katherine Pline, Catherine A. Loynes, Sarah Needs, Maceler Aldrovandi, Jens Tiefenbach, Ewa Bielska, Rachel E. Rubino, Christopher J. Nicol, Robin C. May, Henry M. Krause, Valerie B. O’Donnell, Stephen A. Renshaw, Simon A. Johnston

The following information is missing from the Funding statement: VBO and MA was supported by the European Research Council (Project LipidArrays).

The full Funding statement should now read:

RJE was supported by a British Infection Association postdoctoral fellowship (https://www.britishinfection.org/). SAJ and KP was supported by Medical Research Council and Department for International Development Career Development Award Fellowship MR/J009156/1 (http://www.mrc.ac.uk/). SAJ was additionally supported by a Krebs Institute Fellowship (http://krebsinstitute.group.shef.ac.uk/), Medical Research Foundation grant R/140419 and Medical Research Council Center grant (G0700091). SAR and CL were supported by a Medical Research Council Programme Grant (MR/M004864/1) (http://www.mrc.ac.uk/). RCM, EB, SN and RJE were supported by a Lister Fellowship (www.lister-institute.org.uk/), Medical Research Council Project Grant (MR/J008176/1) (http://www.mrc.ac.uk/). RCM, EB and SAJ were additionally supported the European Research Council Consolidator Award “MitoFun” (https://erc.europa.eu/). HMK and JT were supported by a Canadian Institutes of Health Research, grant # OME-142884, http://www.cihr-irsc.gc.ca/. VBO and MA were supported by a Wellcome Trust grant 094143/Z/10/Z https://wellcome.ac.uk/funding and the European Research Council (Project LipidArrays). The funders had no role in study design, data collection and analysis, decision to publish, or preparation of the manuscript.
