# Supplementary figures and images for: 15-keto-prostaglandin E2 activates host peroxisome proliferator-activated receptor gamma (PPAR-γ) to promote Cryptococcus neoformans growth during infection
Source: PLoS Pathog. 2019 Mar 28;15(3):e1007597. doi: 10.1371/journal.ppat.1007597 (PMC6438442; doi:10.1371/journal.ppat.1007597)

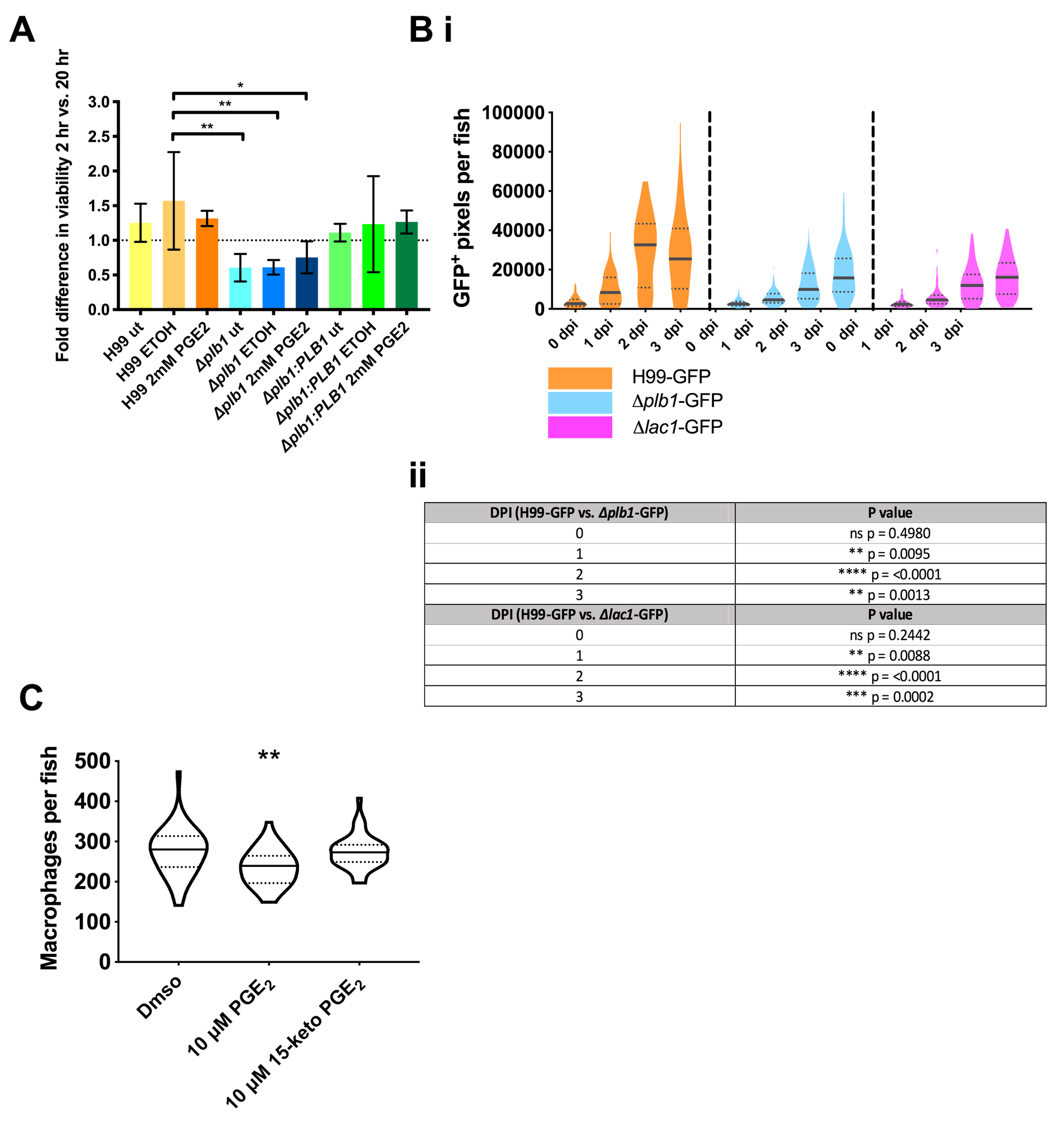

Supplement: S1 Fig — A Quantification of the vialibility of C. neoformans retrieved from the phagosomes of J774 macrophages at 2 hr and 20 hr post infection. Prior to, and during infection J774 macrophages were treated with 2 mM PGE2 or the equivalent amount of solvent (Ethanol) Cryptococcus cells were counted with a hemocytometer / diluted and plated to give an expected number of 200 CFU—dead Cryptococcus cells are indistinguishable from live cells when counting with a hemocytometer however a lower than expected CFU count would indicate that there is a decrease in viability. Data is displayed as the fold change between the CFU count at 2 hpi and 20 hpi for each condition. A one-way ANOVA with Tukey post test was performed comparing all conditions. H99 ETOH vs. Δplb1 ** p = 0.0052, H99 ETOH vs. Δplb1 ETOH ** p = 0.0056, H99 ETOH vs. Δplb1 2 mM PGE2 * p = 0.029. B i Comparison of fungal burden between H99-GFP, Δplb1-GFP and Δlac1-GFP infected larvae (Data reproduced from Fig 2Ai and 2Di and S2Bi Fig for clarity) H99-GFP, Δplb1-GFP and Δlac1-GFP infected larvae imaged at 0, 1, 2 and 3 dpi. At least 50 larvae measured per time point from 3 biological repeats. Box and whiskers show median, 5th percentile and 95th percentile. Unpaired Mann-Whitney U tests used to compare the burden between each strain for every time point. B ii Table of Mann-Whitney U tests comparing burden for each strain between time points. C Whole body macrophage counts of zebrafish larvae treated at 2 dpf with 10 μM PGE2, 10 μM 15-keto-PGE2 or an equivalent DMSO control for 2 days. Box and whiskers show median, 5th percentile and 95th percentile. At least 12 larvae quantified per treatment group per biological repeat n = 4. Mann-Whitney U test used to treatments to DMSO control ** p = 0.0025. (TIF) [file ppat.1007597.s001.tif]

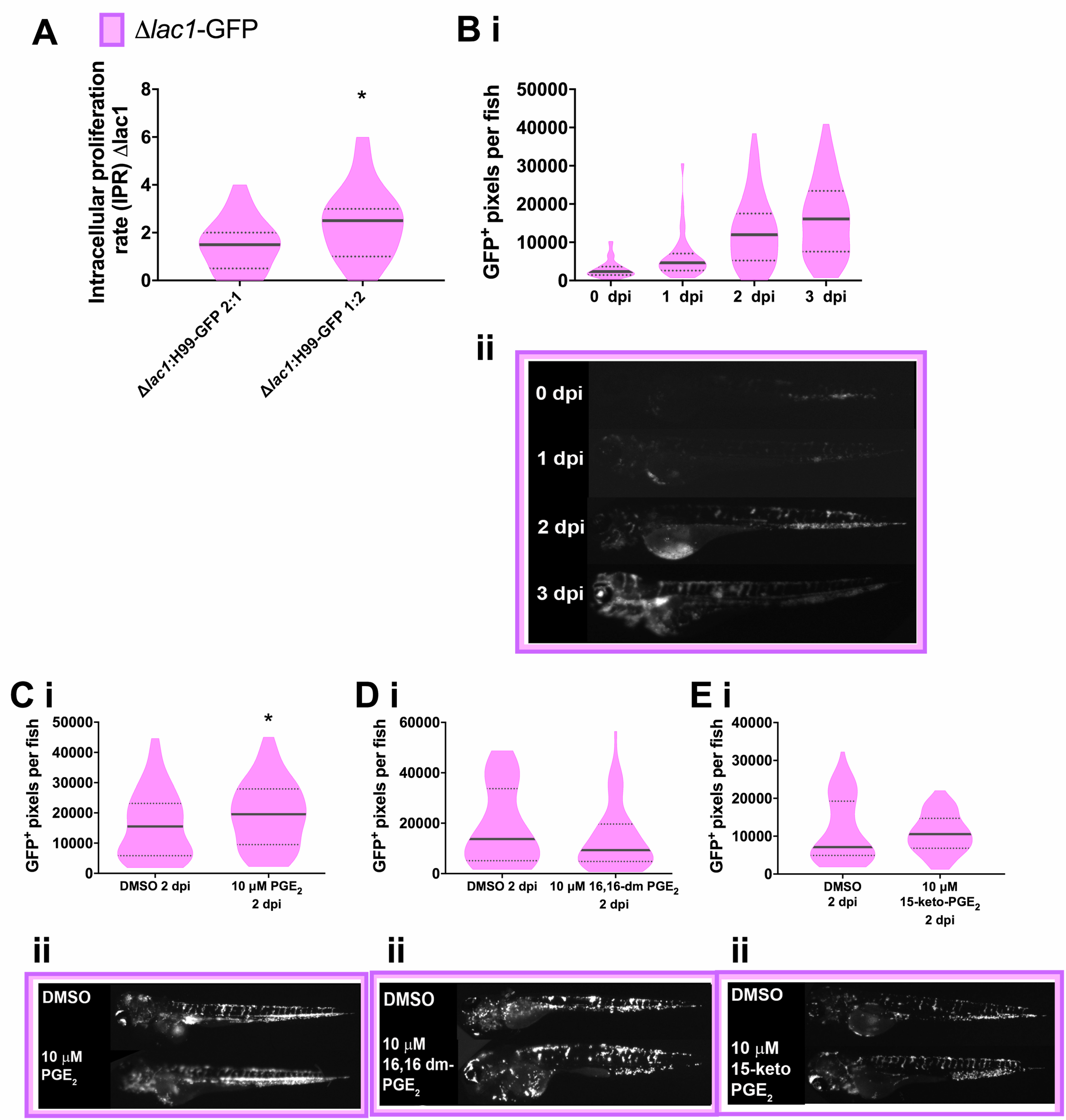

Supplement: S2 Fig — A J774 cells co-infected with a 50:50 mix of Δlac1-GFP and H99. Quantification of IPR for Δlac1-GFP cells within Δlac1-GFP:H99 2:1 or 1:2 co-infected macrophages. At least 20 co-infected macrophages were analysed for each condition over 4 experimental repeats. Student’s T test performed to compare ratios– 2:1 vs 1:2 * p = 0.012. B i Δlac1-GFP infected larvae imaged at 0, 1, 2 and 3 dpi. Fungal burden measured by counting GFP positive pixels in each larvae. At least 78 larvae measured per time point across 3 biological repeats. Box and whiskers show median, 5th percentile and 95th percentile. Unpaired Mann-Whitney U tests used to compare the burden between each strain for every time point, for p values see (S2A and S2B Fig). B ii Representative GFP images (representative = median value) of 2dpi Δlac1-GFP infected larvae, untreated at 0,1,2,3 dpi C i Δlac1-GFP Infected larvae treated with 10 μM prostaglandin E2 or equivalent solvent (DMSO) control. At least 70 larvae measured per treatment group across 4 biological repeats. Box and whiskers show median, 5th percentile and 95th percentile. Unpaired Mann-Whitney U test used to compare between treatments, DMSO vs. 10 μM PGE2 * p = 0.035. D i Δlac1-GFP Infected larvae treated with 10 μM 16,16-dimethyl prostaglandin E2 or equivalent solvent (DMSO) control. At least 75 larvae measured per treatment group across 4 biological repeats. Box and whiskers show median, 5th percentile and 95th percentile. Unpaired Mann-Whitney U test used to compare between treatments, DMSO vs. 10 μM 16,16-dimethyl prostaglandin E2 ns p = 0.062. E i Δlac1-GFP Infected larvae treated with 10 μM 15-keto-prostaglandin E2 or equivalent solvent (DMSO) control. At least 58 larvae measured per treatment group across 3 biological repeats. Unpaired Mann-Whitney U test used to compare between treatments DMSO vs. 10 μM 15-keto-prostaglandin E2 ns p = 0.50. C ii, D ii, E ii Representative GFP images (representative = median value) Δlac1-GFP infected larvae, at [file ppat.1007597.s002.tif]

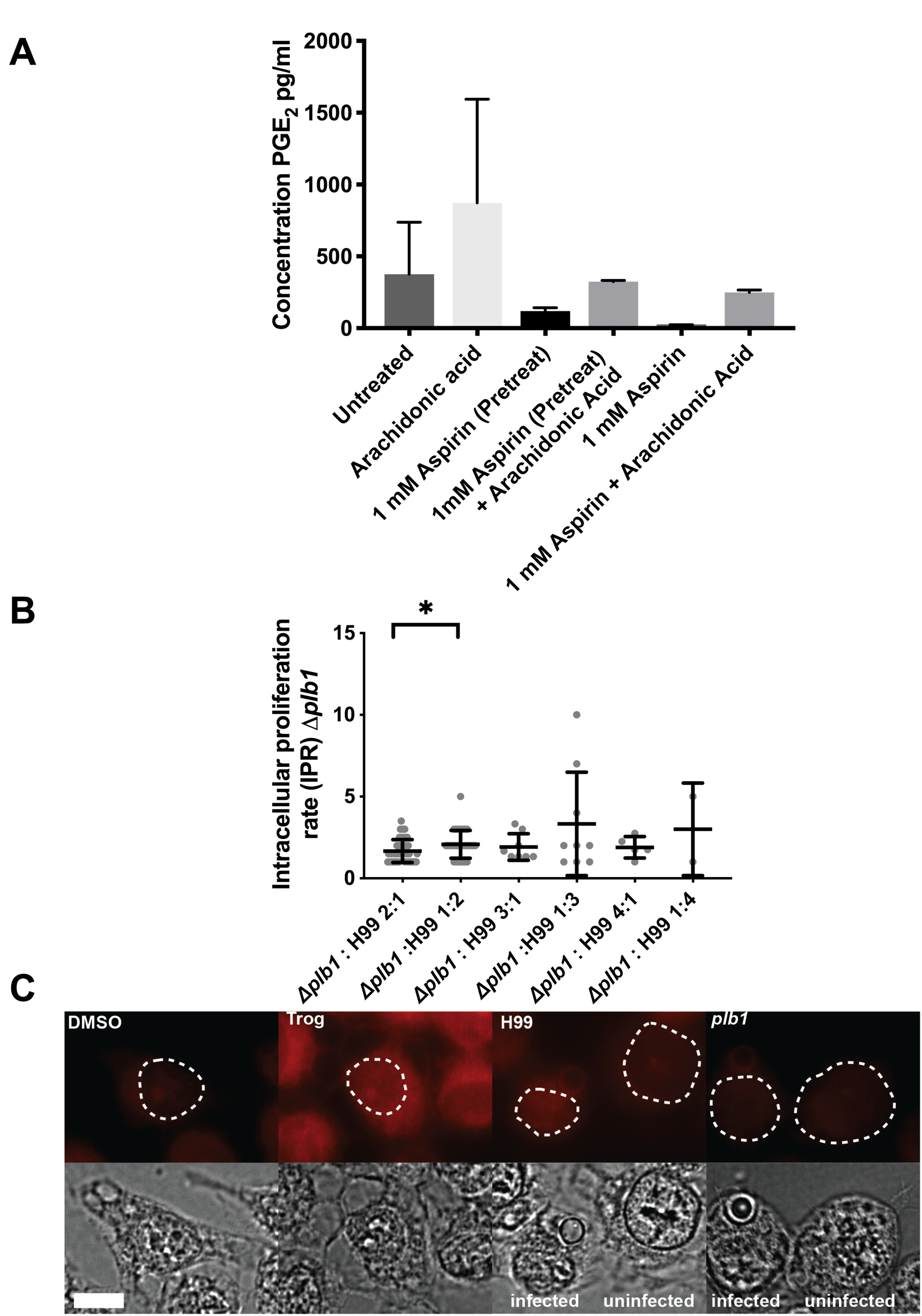

Supplement: S3 Fig — A PGE2 monoclonal EIA ELISA performed on supernatants from C. neoformans infected macrophages collected at 18 hr post infection. Mean concentration of PGE2 (pg per 1x10^6^ cells) plotted with SD, n = 2. B Quantification of IPR for Δplb1 cells within Δplb1:H99-GFP co-infected macrophages at initial burdens of 2:1, 3:1, 4:1 and vice versa. N = 4. Student’s T test performed to compare ratios– 2:1 vs 1:2 * p = 0.0137. C Example images of immunofluorescence experiments in J774 macrophages staining for PPAR-gamma nuclear localization (for quantification see Fig 5Ai). J774 cells treated with DMSO, 0.25 μM Troglitazone, infected with H99-GFP or Δplb1-GFP at x60 magnification, scale bar = 10 μM. Images provided are from the Cy3 channel (PPAR- γ) and the corresponding cell in DIC. The area of the nuclei is marked with a white dotted line. (TIF) [file ppat.1007597.s003.tif]

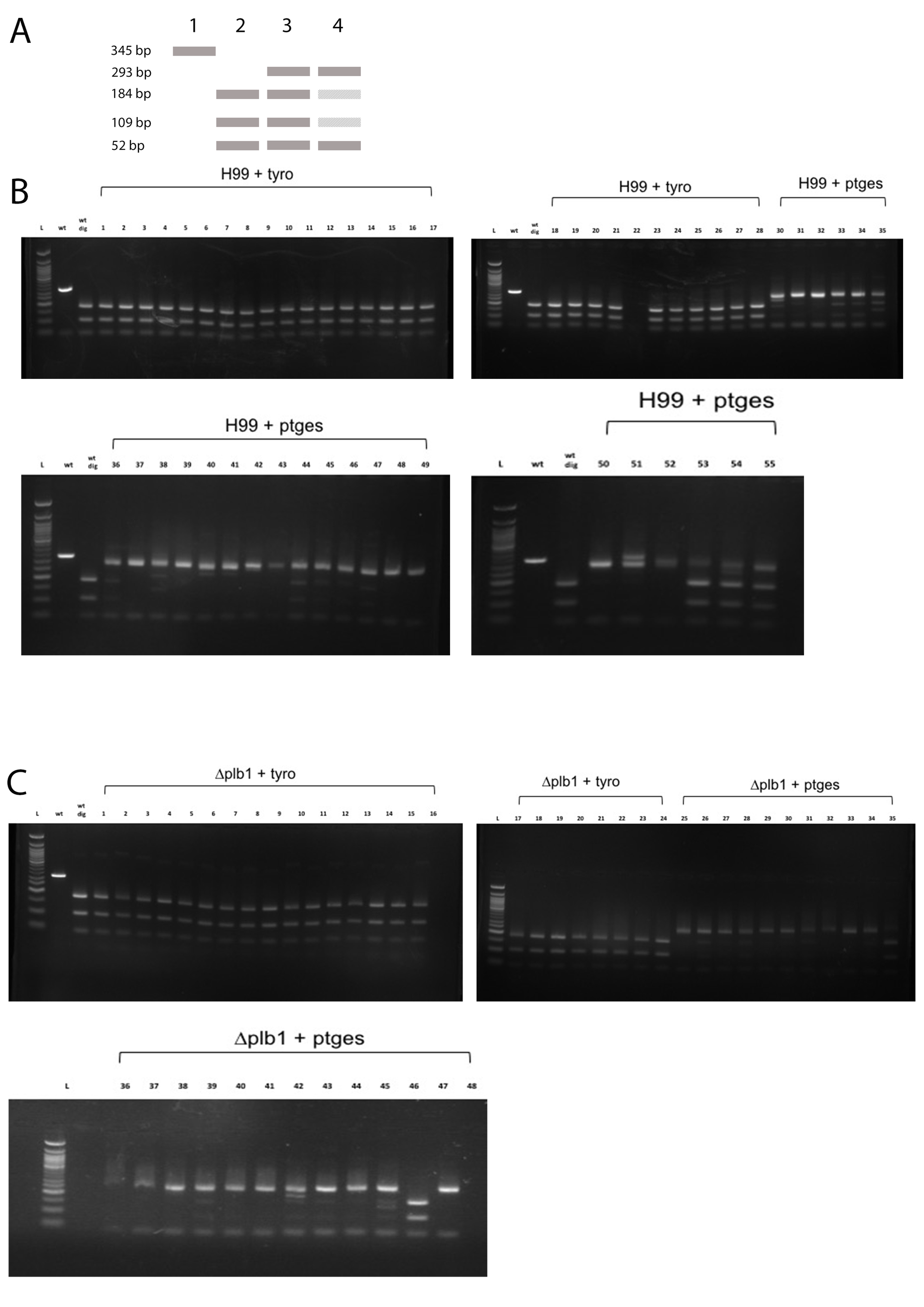

Supplement: S4 Fig — Zebrafish were genotyped post assay (5 dpf), an area of genomic DNA spanning the ptges gene ATG site (the CRISPR target) was amplified with PCR to produce a 345 bp product. This product was digested with Mwol to produce genotype specific banding patterns A. Schematic of the banding patterns expected for each genotype following Mwol digestion– 1. Undigested product, a single 345 bp band 2. Wild type genotype, 184, 109 and 52 bp bands 3. Hetrozygous genotype (ptges +/-) 293, 184, 109 and 52 bp bands 4. Homozygous genotype (ptges -/-) strong bands for 293 and 52 bp, weaker bands at 184 and 109 bp can sometimes be seen indicating a small amount of wildtype ptges is still present (this is thought to be beneficial as low levels of ptges are required for larvae survival. B Genotyping for H99 infected larvae, L = DNA ladder (NEB 50 bp ladder), wt = undigested wild type control, wt dig = wild type digested control, numbers correspond to individual larvae genotyped. H99 + tyro = tyr -/- larvae infected with H99-GFP. H99 + ptges = ptges -/- larvae infected with H99-GFP C Genotyping for Δplb1 infected larvae, L = DNA ladder (NEB 50 bp ladder), wt = undigested wild type control, wt dig = wild type digested control, numbers correspond to individual larvae genotyped. Δplb1+ tyro = tyr -/- larvae infected with Δplb1-GFP. Δplb1+ ptges = ptges -/- larvae infected with Δplb1-GFP. (TIF) [file ppat.1007597.s004.tif]
